# FcαRI Dynamics Are Regulated by GSK-3 and PKCζ During Cytokine Mediated Inside-Out Signaling

**DOI:** 10.3389/fimmu.2018.03191

**Published:** 2019-01-31

**Authors:** Toine ten Broeke, Henk Honing, Arianne M. Brandsma, Shamir Jacobino, Jantine E. Bakema, Deon Kanters, Jan A. M. van der Linden, Madelon Bracke, Leo Koenderman, Jeanette H. W. Leusen

**Affiliations:** ^1^Laboratory of Translational Immunology, University Medical Center, Utrecht, Netherlands; ^2^Department of Respiratory Medicine, University Medical Center, Utrecht, Netherlands; ^3^Tumor Biology Section, Department of Otolaryngology, Head-Neck Surgery, VU University Medical Center, Amsterdam, Netherlands; ^4^Department of Pharmacoepidemiology and Pharmacotherapy, University Medical Center, Utrecht, Netherlands

**Keywords:** Fc alpha receptor I, IgA, glycogen synthase kinase-3, Protein Kinase C zeta, fluorescence recovery after photobleaching

## Abstract

IgA binding to FcαRI (CD89) is rapidly enhanced by cytokine induced inside-out signaling. Dephosphorylation of serine 263 in the intracellular tail of FcαRI by PP2A and PI3K activation are instrumental in this process. To further investigate these signaling pathways, we targeted downstream kinases of PI3K. Our experiments revealed that PI3K activates PKCζ, which subsequently inhibits GSK-3, a constitutively active kinase in resting cells and found here to be associated with FcαRI. We propose that GSK-3 maintains FcαRI in an inactive state at homeostatic conditions. Upon cytokine stimulation, GSK-3 is inactivated through a PI3K-PKCζ pathway, preventing the maintenance of phosphorylated inactive FcαRI. The concomitantly activated PP2A is then able to dephosphorylate and activate FcαRI. Moreover, FRAP and FLIP studies showed that FcαRI activation coincides with an increased mobile fraction of the receptor. This can enhance FcαRI valency and contribute to stronger avidity for IgA immune complexes. This tightly regulated inside-out signaling pathway allows leukocytes to respond rapidly and efficiently to their environment and could be exploited to enhance the efficacy of future IgA therapeutics.

## Introduction

Transmembrane receptors specific for the Fc-portion of immunoglobulins, Fc-receptors (FcR), play an important role in leukocyte activation by recognizing and binding of opsonized targets during inflammatory processes (1). Specific FcR exist for all 5 classes of human immunoglobulins of which those specific for IgG and IgE are best studied. Less is known about the receptor specific for monomeric IgA, FcαRI (CD89). FcαRI is expressed on many cell types, including monocytes/macrophages, neutrophils, and eosinophils (2, 3). FcR play a major role in effector mechanisms induced by many IgG therapeutics currently used in the clinic (4, 5). Next to IgG, promising potential of therapeutic IgA monoclonals found in preclinical studies implicate that their efficacy largely depend on FcαRI, validating the need for more knowledge on FcαRI function (6–9).

In this study, we investigated the regulation of FcαRI by inside-out signaling. Inside-out signaling refers to a process where stimulation of a cell (e.g., by cytokines) results in increased binding of a certain ligand, without changing expression levels of its receptor at the cell surface (3). This advocates that the affinity and/or avidity of a receptor is regulated by inside-out signaling. A well described example is the conformational change of integrins after cytokine stimulation, resulting in increased affinity for ligand (10). The increase of ligand binding capacity or activation of FcαRI, FcγRI (CD64), and FcγRII (CD32) on primary human leukocytes is also regulated by cytokines (11–14). Furthermore, FcαRI activation is induced by protein phosphatase 2A (PP2A) after it dephosphorylates a single serine residue (S263) in the FcαRI intracellular tail (15, 16). Previous work demonstrated that signaling through phosphatidylinositol 3-kinase (PI3K) is critical for the cytokine induced FcαRI activation, which depends on its associated FcR γ-chain (16, 17). Upon activation of PI3K at the membrane, PI(3,4,5)P3-dependent kinases (PDKs) are recruited to the membrane and phosphorylate PI3K effectors such as Protein Kinase B (PKB)/Akt (18), p70 S6 Kinase (p70S6K) (19), and Protein Kinase C (PKC)-isoforms including the atypical PKCζ (20–22).

We further investigated the cytokine regulated mechanism that activates FcαRI, using an IL-3 dependent murine pre-B cell (Ba/F3) model system (16, 23). Since PI3K is critical for cytokine induced FcαRI activation, we focused on signaling pathways downstream of PI3K. We show that PI3K exerts its role in FcαRI modulation by activating PKCζ, which is then able to inhibit glycogen synthase kinase-3 (GSK-3). GSK-3 is constitutively active in the absence of cytokine stimulation and can phosphorylate S263, keeping FcαRI in the inactive state. As a result of PKCζ activation and subsequent GSK-3 inhibition, the concomitantly upregulated PP2A dephosphorylates S263 and activates the FcαRI (15). Finally, we show by fluorescence recovery after photobleaching (FRAP) and fluorescence loss in photobleaching (FLIP) that the amount of mobile FcαRI at the plasma membrane is influenced by cytokine stimulation. This process of cytokine induced inside-out signaling is similar to that observed in the regulation of integrins (24). In this way leukocytes are able to respond quickly and efficiently to immunological cues and optimally perform their function when required.

## Materials and Methods

### Reagents, Antibodies, and Incubation Buffer

Purified human serum IgA (>20 mg/ml) was obtained from Cappel (Malvern, PA). It contained no detectable trace of IgG, IgM, or non-immunoglobulin serum proteins. Recombinant mouse IL-3 was produced in COS cells (25). Ultra-pure fibrinogen (Fib3) and human alpha thrombin was purchased from Enzyme Research Laboratories (Swansea, UK). Pharmacological inhibitors LY294002, SB216763 were purchased from BioMol (Plymouth Meeting, PA). PKB inhibitor 1L-6-Hydroxymethyl-*chiro*-inositol 2-(R)-2-O-methyl-3-O-octadecylcarbonate was obtained from Calbiochem (San Diego, CA), the GSK-3α/β inhibitor CHIR-99021 from Selleckchem and okadaic acid from Enzo Life Sciences. The PKCζ pseudo-substrate was purchased from Biosource (Camarillo, CA) or Santa Cruz biotechnology. Recombinant human GM-CSF (rhGM-CSF) was from Immunotools. PI-3 kinase construct p110-K227E was a kind gift of Dr. J. Downward (ICRF, London, UK). G418 and hygromycin B were purchased from Boehringer Mannheim (Germany). Incubation buffer contained 20 mM HEPES, 132 mM NaCl, 6 mM KCl, 1 mM MgSO_4_, 1.2 mM KH_2_PO_4_, supplemented with 5 mM glucose, 1 mM CaCl_2_, and 0.5% (w/v) HSA. All other materials were reagent grade.

### Cell Lines and Generation of Stable Transfectants

Ba/F3 cells were cultured at a cell density of 10^5^-10^6^ cells/ml in RPMI 1640 supplemented with 8% Hyclone serum (Gibco, Rockville, MD) and require IL-3 for their survival and proliferation. The FcαRI wt and mutant cell lines have been generated as described before (17) and cultured in the presence of 500 μg/ml hygromycin B. For the generation of polyclonal transfectants, pMT2 containing GSK-3β wt, GSK-3β S9A, PKCζ wt, or PKCζ kinase dead were electroporated into Ba/F3 cells (0.28 V; capacitance 960 μFD) together with LXSN-neo. Stable cell lines were grown continuously in the presence of G418 and hygromycin B. Monocytes from the blood of healthy donors were freshly isolated from the PBMC fraction obtained from a standard ficoll gradient centrifugation protocol, by using CD14-microbeads according to the manufactures protocol (Miltenyi Biotec) and immediately used.

### FcαRI-YFP Fusion Constructs

Human FcαRI(wt) (16) and pYFP-C1 (Clontech, Mountain View, CA) were used as a template and restriction sites were added via PCR using the following primers: FcαRI(wt) (F_wt_: CCGGGGGAGGCACAGATCTTGGAAGG and R_wt_: TAAAGCGGCCGCACTTGCAGACACTTGGTGT), FcαRI(S263A) (F_wt_ and R_sa_: TAAAGCGGCCGCACTTGCAGACAGCTGGTGT), FcαRI(S263D) (F_wt_ and R_sd_: TAAAGCGGCCGCACTTGCAGCTGGTGT) and YFP (F_yfp_: ATAAGAGGCCGCATGGTGAGCAAGGGCGAG and R_yfp_: TGCTCTAGATTATCCGGACTACAGCTC). FcαRI(wt) and FcαRI-mutants were ligated in the pEGFP-N1 vector (Clontech Mountain View, CA) using the restriction sites SmaI, NotI, and XbaI, thereby deleting the internal EGFP. All constructs were verified by sequencing. For the generation of polyclonal transfectants, pEGFP-N1 containing FcαRI(wt)-YFP, FcαRI(S263A)-YFP or FcαRI(S263D)-YFP were electroporated into Ba/F3 cells (0.28 V; capacitance 960 μFD). Stable cell lines were maintained in medium containing G418.

### IgA Binding Assays

The IgA binding assays were performed with cytokine-starved Ba/F3 cells unless indicated otherwise. For IL-3 starvation, Ba/F3 cells were washed twice with phosphate-buffered saline (PBS) and left in IL-3 free medium (RPMI 1640, containing 0.5% serum) for 4 h. Prior to performing a binding assay, Ba/F3 cells were washed with Ca^2+^-free incubation buffer containing 0.5 mM EGTA and brought to a concentration of 8 × 10^6^ cells/ml. A 50 μl cell suspension (0.4 × 10^6^ cells) was pre-incubated at 37°C with or without IL-3 for 15 min. After stimulation of the cells, Dynabeads coated with serum IgA (10 mg/ml) as described previously (12) were added in a ratio of 3.5 beads/cell. After brief mixing, cells and beads were pelleted for 15 s at 100 rpm and incubated for 30 min at 37°C. Subsequently, cells were suspended vigorously and IgA binding was evaluated under a microscope. One hundred cells were scored, and the number of beads that were bound to the cells was counted. The amount of beads bound to a total of 100 cells (bound and unbound to beads) was designated as the rosette index. As described previously, the rosette method was specific because there is no appreciable background binding of cells to beads coated with ovalbumin (23, 26). Binding of IgA to freshly isolated monocytes was adapted from Bakema et al. (15). In brief, monocytes suspended in PBS 0,5% BSA were first allowed to adhere for 30 min at 37°C to a 96-well flat bottom plate in the presence or absence of 1 μM OA, washed and followed by a 15 min incubation at 37°C with or without 1 μM OA, 10 ng/ml rhGM-CSF or 10 μM PKCζ pseudo-substrate or a combination of these. Then, Dynabeads coated with an in-house produced anti-CD20 IgA_2_ (coating was tested by RPE labeled anti-IgA in flow cytometry) were added in a ratio of 3.5 beads/cell, mixed, spun down and allowed to bind the monocytes for 10 min at 37°C. Cells were then washed once with PBS, fixed in 3% paraformaldehyde for 15 min and at least five images per condition were taken using bright-field microscopy (EVOS^®^XLCore) for rosette quantification.

### Inhibition of IgA Binding With Pharmacological Inhibitors or Peptides

For inhibition studies, cytokine-starved cells were pre-incubated with specific inhibitors prior to incubation with IL-3. Cells were incubated with PI-3K inhibitor LY294002 for 15 min at a final concentration of 1 μM. The GSK-3 inhibitor SB-216763 was incubated for 15 min at the indicated concentrations. The PKCζ pseudo-substrate was used at the indicated concentrations for 10 min prior to cytokine stimulation.

### GSK-3 Phosphorylation

Ba/F3 cells were washed twice with PBS and incubated in medium without IL-3 (RPMI 1640 with 0.5% serum) for 4 h. To investigate the effect of the PKCζ pseudo-substrate on GSK-3 phosphorylation after IL-3 stimulation, cells were stimulated for 20 min at 37°C with IL-3 with or without pre-incubation of 10 min with the PKCζ pseudo substrate (10 μM) or the PI3K inhibitor LY294002 (1 μM). For detection of phosphorylation of GSK-3, Ba/F3 cells (2 × 10^6^ per condition) were washed twice in ice-cold PBS after stimulation and solubilized in lysis buffer (1% Triton-X100, 50 mM Tris-HCl, pH 8.0, 100 mM NaCl) with protease/phosphatase inhibitor (1 μg/ml leupeptin, 1 mM PMSF, 10 μg/ml aprotinin, 1 mM sodiumorthovanadate, and 0.5 mM benzamidine). Subsequently, 5x Laemmli sample buffer was added and the lysates were boiled for 5 min. Total cell lysates were analyzed on 12% SDS-polyacrylamide gels. Proteins were transferred to Immobilon-P and incubated with blocking buffer (Tris-buffered saline/Tween20 supplemented with 1 mM EDTA and 5% bovine serum albumin) containing phospho-GSK-3 (Ser 9/21, Cell Signaling Technology Inc., Danvers, MA) or total-GSK-3 (Upstate, Lake Placid, NY) antisera. Detection was achieved using enhanced chemiluminescence (ECL, Amersham, UK).

### *In vitro* Kinase Assay

*In vitro* phosphorylation of GST-FcαRI intracellular domain fusion proteins was performed as described previously in Bracke et al. (16). However, recombinant GSK-3β (ITK diagnostics BV, Moutain view, CA) was used instead of cell lysates as kinase source. Briefly, 10 μg of GSK-3β was incubated with GST-FcαRI intracellular domain fusion proteins or GST proteins alone, in kinase buffer (25 mM Tris-Hcl pH 7.5, 25 mM MgCl_2_, 50 μM ATP, 3 μCi γ^32^P-ATP), and incubated for 30 min at RT. Samples were washed before addition of 5x Laemmli sample buffer and analyzed by electrophoresis on 15% SDS-polyacrylamide gels. Substrate phosphorylation was detected by autoradiography.

### Confocal Microscopy, Fluorescence Recovery After Photobleaching (FRAP), and Fluorescence Loss in Photobleaching (FLIP)

FcαRI-YFP wt or S263 mutant expressing Ba/F3 cells were washed once with PBS and once with medium (RPMI 1640 without phenol red (Gibco) supplemented with 1% FCS and 2 mM L-glutamin). The cells were then seeded in a μ-Dish (35 mm, high; Ibidi) at 4°C in a fibrin-matrix (2.5 mg/mL fibrinogen and 1 × 10^−4^ U/uL thrombin in medium) and incubated at 37°C. After matrix formation, extra medium was added on top and the cells were incubated overnight. IL-3 was always omitted from the medium 16–20 h before the start of the experiments. Next day, FRAP measurements were performed with or without 20 min IL-3 stimulation. The cells were pre-incubated for 30 min with inhibitors before IL-3 stimulation and/or FRAP measurements. FRAP experiments were performed on a Zeiss LSM710 confocal microscope with a 63x oil objective lens equipped with an environmental chamber for temperature (37°C) and CO_2_ (5%) control. To study FcαRI lateral mobility, we adapted a protocol using strip-FRAP (27, 28). An argon laser provided the 488 nm excitation. Ten pre-bleach images were acquired, after which a small square area (~1 μm^2^) spanning the membrane was bleached for 0.2 s to obtain a bleach of ~50%. The fluorescence in this region was monitored by acquiring images at 7.4 frames/s for 30–35 s per cell. For each condition, >100 cells were measured.

The fluorescence recovery of the bleached area was calculated using a script written in MATLAB (MathWorks, Inc., Natick, USA). Within this script the fluorescence intensity of the bleached strip was corrected for loss of fluorescence during the measurement (by subtracting the background fluorescence intensity and correcting for the overall fluorescence intensity) and normalized (by setting the mean fluorescence before bleaching to 1; this corrects for differences in cell fluorescence between measurements). The relative mean fluorescence intensities of the bleached strip of all cells for each condition were plotted and non-linear two-phase association (GraphPad Prism 7 software) was used to fit the experimental data. To determine the mobile fraction (*M*_*f*_) of receptors, the following equation was used for the normalized data:

$$\begin{array}{r} M_{f}=\frac{I_{plateau}-I_{0}}{1-\;I_{0}}\times 100\% \end{array}$$

where *I*_*plateau*_ is the maximal fluorescence intensity of the two-phase association fit (plateau) and I_0_ is the average fluorescence intensity directly after bleaching (minimum y-axis value of the two-phase association fit). The recovery t½ was calculated in GraphPad by two phase association curve fitting when *I*_0_ was set to zero.

For FLIP, cells were washed and suspended in medium [RPMI 1640 without phenol red (Gibco) supplemented with 1% FCS and 2 mM L-glutamin] and seeded on glass coverslips (Labtek II, Nalge Nunc) in a fibrin matrix. IL-3 stimulated and non-stimulated cells were imaged using a Zeiss LSM 510 Meta confocal microscope as follows: a sequence of 20–30 images was made and in between images, bleaching of a small part of the cell spanning the plasma membrane was performed by maximal laser power. The time course of lost fluorescence was monitored at the other side of the cellular image with Optimas Image Analysis software (Optimas Corp. Bothell, Washington, USA), taking into account background bleaching during the imaging sequence that was determined on neighboring cells which were not bleached. FLIP measurement of FcαRI S263A/D mutant expressing cells were performed after IL-3 stimulation. For FLIP measurements displayed in Supplemental Figure 4, FcαRI-YFP wt expressing Ba/F3 cells were seeded in a fibrin matrix in the same way as for the FRAP measurements. FLIP was then measured live on a Zeiss LSM710 confocal microscope with a 63x oil objective lens equipped with an environmental chamber for temperature (37°C) and CO_2_ (5%) control. The fluorescence loss was calculated by subtracting the background fluorescence intensity and correcting for the overall loss of fluorescence intensity during the repetitive bleaching cycles. The data was then normalized by setting the mean fluorescence from six images before bleaching to 100%. The relative loss in fluorescence intensities for each condition were plotted and non-linear one-phase association (GraphPad Prism 7 software) was used to apply curve fitting of the experimental data.

### Statistical Analysis

Results of the IgA binding assays are expressed as mean ± SD. Statistical analysis was performed by using paired Students *t*-tests or repeated measures ANOVA, *p* < 0.05 were considered as statistically significant. Best curve fitting for the FRAP analyses was performed in GraphPad Prism and confirmed by *F*-test.

## Results

### PKCζ Is Involved in Cytokine Induced FcαRI Activation

**Figure 1:**
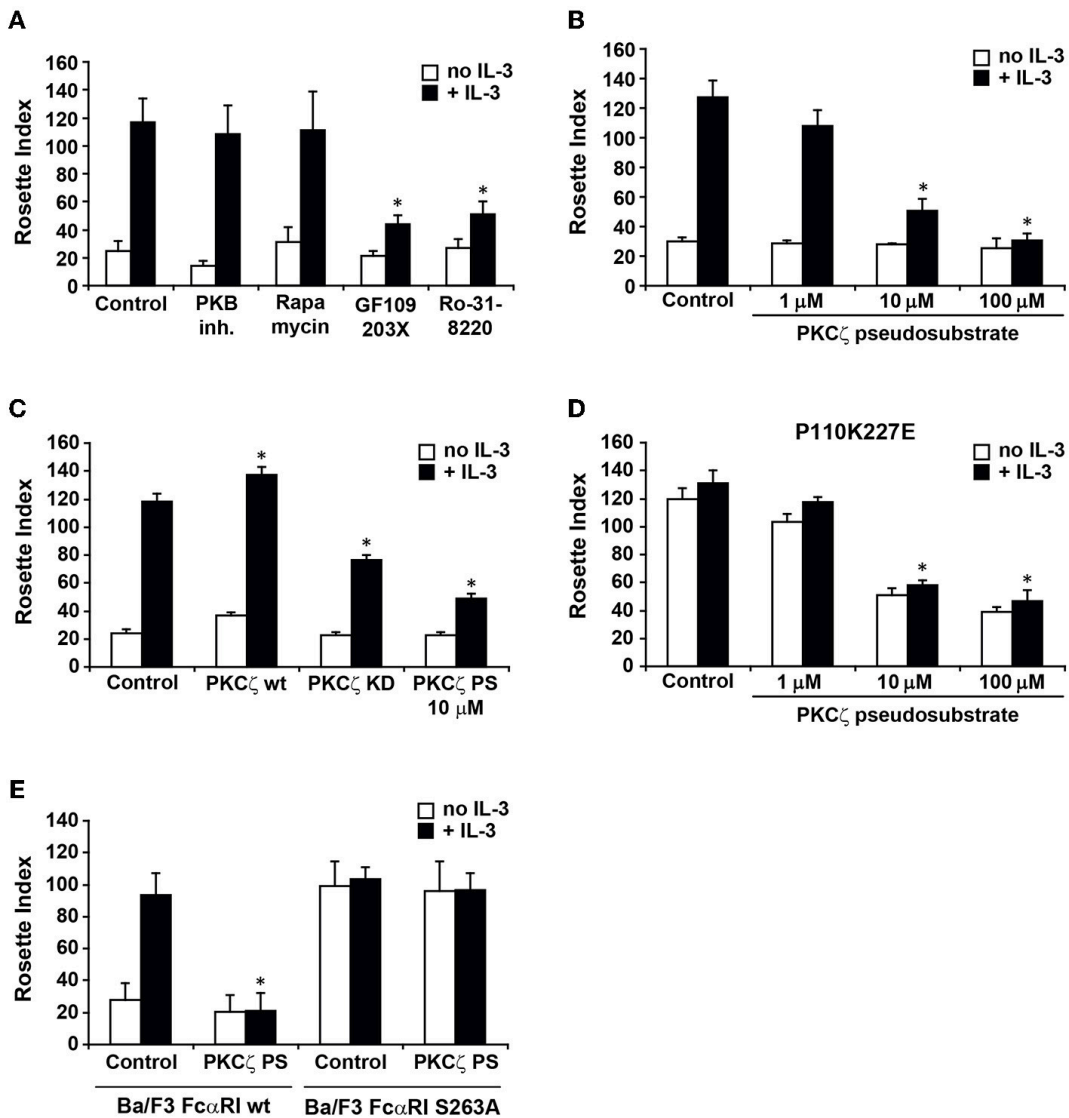
Involvement of PKCζ in cytokine induced FcαRI activation. **(A)** Cytokine starved Ba/F3 FcαRI cells were pre-treated for 15 min at 37°C with PKB inhibitor, rapamycin (a p70S6K inhibitor) or PKC inhibitors GF109203X (1 μM) and Ro31-8220 (1 μM). **(B)** Ba/F3 FcαRI or **(D)** Ba/F3 FcαRI(p110K227E) were pre-treated for 15 min with indicated concentrations of PKCζ pseudo-substrate. **(C)** Ba/F3 FcαRI cells overexpressing PKCζ wt or PKC kinase dead (KD) mutant were cytokine starved. **(E)** Cytokine starved Ba/F3 FcαRI wt or Ba/F3 FcαRI S263A cells were pretreated with 10 μM PKCζ pseudo-substrate (PKCζ PS) for 15 min. All cells were then stimulated with or without IL-3 for 15 min at 37°C. Number of IgA-beads to these cells was measured and IgA binding is expressed as the mean rosette index (number of beads/100 cells) ± SD (*n* = 3). Values indicated with ^*^differed significantly (*p* < 0.05) from the control.

We have previously demonstrated that IgA binding to FcαRI on human eosinophils is modulated by cytokine induced activation of PI3K (17). In addition, the phosphorylation status of a single C-terminal serine (S263) dictates FcαRI activity (16). To further investigate which pathways could be involved in cytokine induced FcαRI activation, we used pharmacological inhibitors of known targets of PI3K, like p70 S6 Kinase (p70S6K), PKB, and PKC isoforms, in a rosette assay. As shown in Figure 1A, FcαRI wt expressing Ba/F3 cells exhibited some residual binding of IgA beads when IL-3 stimulation was absent. In contrast, addition of IL-3 to these cells resulted in an increase of IgA binding. Treatment with either a PKB inhibitor or rapamycin, which inhibits p70S6K activation, did not affect IgA binding (17) (Figure 1A). Interestingly, treatment with either GF109203X or Ro-31-8220, both PKC inhibitors, resulted in a decrease in IgA binding to IL-3 stimulated cells [Figure 1A and (17)]. We continued by investigating whether the atypical PKC isoform, PKCζ, could be responsible for the inhibition by the general PKC inhibitors. PKCζ was the most likely candidate to study, because both PKCζ and IgA binding to FcαRI are Ca^2+^ and diacylglycerol (DAG) independent (22). A pseudo-substrate specific for PKCζ inhibited IL-3 induced IgA binding in a dose-dependent manner (Figure 1B). Using this pseudo-substrate we could also confirm the role of PKCζ in cytokine mediated inside-out signaling in freshly isolated monocytes from healthy donors (Supplemental Figure 1). Furthermore, stable expression of a kinase-dead PKCζ resulted in a partial inhibition of IL-3 mediated IgA binding, whereas additional expression of the wild type PKCζ significantly increased the IgA binding (Figure 1C). The PKCζ pseudo-substrate also blocked the binding to Ba/F3 FcαRI cells in which p110K227E was overexpressed, a catalytic subunit mutant that acts as a constitutively active form of PI3K (29) (Figure 1D). We also tested whether the PKCζ pseudo-substrate would affect IgA binding to the constitutive active FcαRI S263A mutant. As expected, the FcαRI S263A mutant was insensitive to PKCζ inhibition, suggesting that the PKCζ pseudo-substrate acted specifically on the cytokine induced activation of FcαRI (Figure 1E). Together, these data show that cytokine induced activation of PKCζ downstream of PI3K is necessary for FcαRI activation.

### GSK-3 Activity Phosphorylates FcαRI and Is Inhibited Downstream of Cytokine Induced PKCζ

We next investigated how the signal from PKCζ could modulate the function of FcαRI. Previous studies suggest that the “default” binding state of the receptor is high, but suppressed by S263 phosphorylation in unstimulated cells (16, 23). Cytokine stimulation may release or overrule this suppression, switching the receptor to a ligand binding state. We hypothesized that, in unstimulated cells, a constitutive active kinase continuously suppresses FcαRI activation by phosphorylating S263, which is crucial in the negative regulation of FcαRI. Cytokine stimulation may lead to inhibition of this kinase through a PI3K-PKCζ dependent pathway.

**Figure 2:**
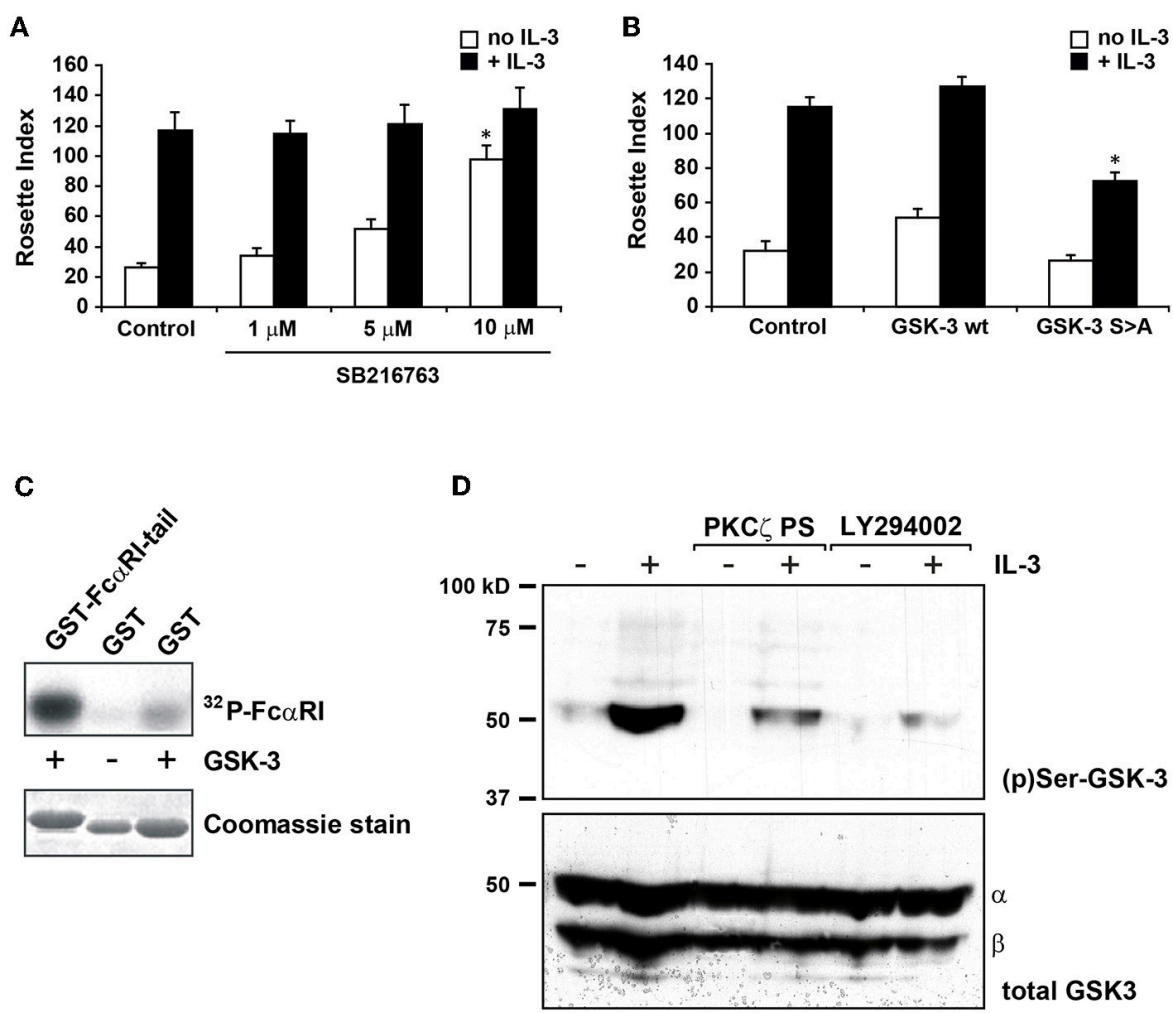
GSK-3 is important in cytokine induced FcαRI activation and operates downstream of PKCζ. **(A)** Cytokine starved Ba/F3 FcαRI cells were pre-treated for 15 min at 37°C with indicated concentrations of GSK-3 inhibitor SB216763 and subsequently stimulated at 37°C with or without IL-3 for 15 min. **(B)** Ba/F3 FcαRI cells co-expressing GSK-3 wt or constitutive active GSK-3 S9A were cytokine starved, and treated with or without IL-3 for 15 min at 37°C. Binding of IgA-beads to these cells was determined and expressed as rosette index (number of beads/100 cells), means ± SD (*n* = 3). Values indicated with ^*^differed significantly (*p* < 0.05) from the control. **(C)** To determine whether GSK-3 can phosphorylate FcαRI, GST-tagged intracellular domain of FcαRI is used for an *in vitro* kinase assay with recombinant GSK-3β. Hereafter, constructs are isolated with glutathione-coated beads and visualized by autoradiography (upper panels) and protein staining for verifying equal loading (lower panels). **(D)** Cytokine starved Ba/F3 FcαRI cells were incubated with PKC**ζ** pseudo-substrate (10 μM) or with the PI-3K inhibitor LY294002 (1 μM) for 10 min. Subsequently, the cells were stimulated with or without IL-3 for 20 min at 37°C, washed with ice-cold PBS, solubilized in Triton-X100 lysis buffer and heated for 5 min after addition of SDS sample buffer. GSK-3 was detected using anti-phospho-GSK-3 or anti-total-GSK-3 antisera revealing that both GSK-3 isoforms are present in the cells.

Glycogen synthase kinase 3 (GSK-3) is a crucial regulator of many cellular functions and is constitutively active in unstimulated cells. Its activity is significantly reduced by phosphorylation of an N-terminal serine, S9 in GSK-3β and S21 in GSK-3α (30). Several kinases can phosphorylate these serines, including PKB, protein kinase A (PKA) but also PKCζ (31, 32). Therefore, we investigated whether GSK-3 could play a role in cytokine induced PI3K-PKCζ dependent FcαRI regulation. In Figure 2A, addition of a specific GSK-3 inhibitor, SB-216763, increased the binding of IgA in cytokine starved Ba/F3 FcαRI cells in a dose dependent manner, suggesting that active GSK-3 in cytokine starved Ba/F3 cells promotes the inactive state of FcαRI.

To further investigate the role of GSK-3 in FcαRI activation, we generated Ba/F3 FcαRI cells overexpressing GSK-3 wt or constitutively active GSK-3 S9A mutant. As shown in Figure 2B, overexpression of GSK-3 wt did not affect IgA binding. However, cytokine induced IgA binding by constitutively active GSK-3 overexpressing cells was significantly reduced, suggesting that GSK-3 contributes to the regulation of ligand-binding to FcαRI.

To investigate whether FcαRI can be phosphorylated by GSK-3, we performed *in vitro* kinase assays with the intracellular domain of FcαRI as a substrate. Recombinant GSK-3 was incubated with the GST-coupled intracellular domain of FcαRI wt or with GST only. As shown in the first lane of Figure 2C, the intracellular tail of FcαRI clearly became phosphorylated by recombinant GSK-3, relative to GST alone. In addition, we performed western blot analysis on whole cell lysates to investigate whether GSK-3, as a downstream target of PI3K and PKCζ, was phosphorylated upon cytokine stimulation and thereby inactivated. GSK-3 was phosphorylated upon stimulation with IL-3 in cytokine starved Ba/F3 cells (Figure 2D). In contrast, the IL-3 induced phosphorylation of GSK-3 was dramatically decreased after pre-incubation with the PKCζ pseudo-substrate or with the PI3K inhibitor LY294002. Together, these results implicate that GSK-3 not only directly phosphorylates the intracellular domain of FcαRI, but it is also subject of phosphorylation and inhibition by the cytokine induced PI3K-PKCζ pathway.

### Cytokine Stimulation Regulates the Mobility of FcαRI at the Plasma Membrane

**Figure 3:**
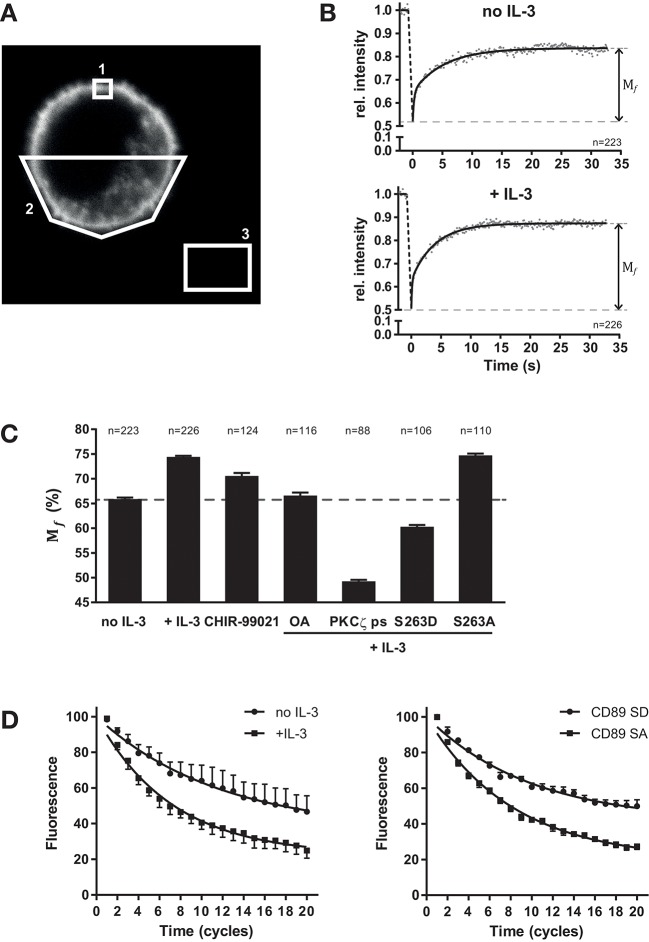
Regulation of FcαRI lateral mobility by cytokine stimulation. FRAP and FLIP measurements of FcαRI-YFP, FcαRI S263A-YFP, or FcαRI S263D-YFP in the absence or presence of IL-3 stimulation and GSK-3 inhibitor CHIR-99021 (5 μM), PP2A inhibitor okadaic acid (1 μM) or PKCζ ps (10 μM). **(A)** Example confocal image of a FcαRI expressing Ba/F3 cell including the regions which were used for FRAP analysis. Region 1 is the bleach area were fluorescence recovery was monitored. Region 2 was used as reference to correct for overall loss of fluorescence intensity during the measurement. Region 3 was used for background subtraction. **(B)** Ba/F3 FcαRI-EYFP wt cells were starved and then stimulated with or without IL-3 before FRAP measurements. Mean values of cells (no IL-3 *n* = 223 cells; + IL-3 *n* = 226 cells) are plotted and *M*_*f*_ of both conditions were determined using two phase association curve fitting. **(C)** Summary of all *M*_*f*_ found for the indicated conditions. For each condition, data of three or more experiments were pooled and used to determine the *M*_*f*_. **(D)** FLIP measurements of FcαRI-YFP in the absence or presence of IL-3 (left) and of the FcαRI S263A-YFP and FcαRI S263D-YFP mutants (right) in the presence of IL-3. Mean of corrected and normalized fluorescence values (±SEM) of cells pooled from four experiments are plotted and one phase association curve fitting was performed using Graphpad 7. Fluorescence before the start of FLIP was set at 100%.

**Figure 4:**
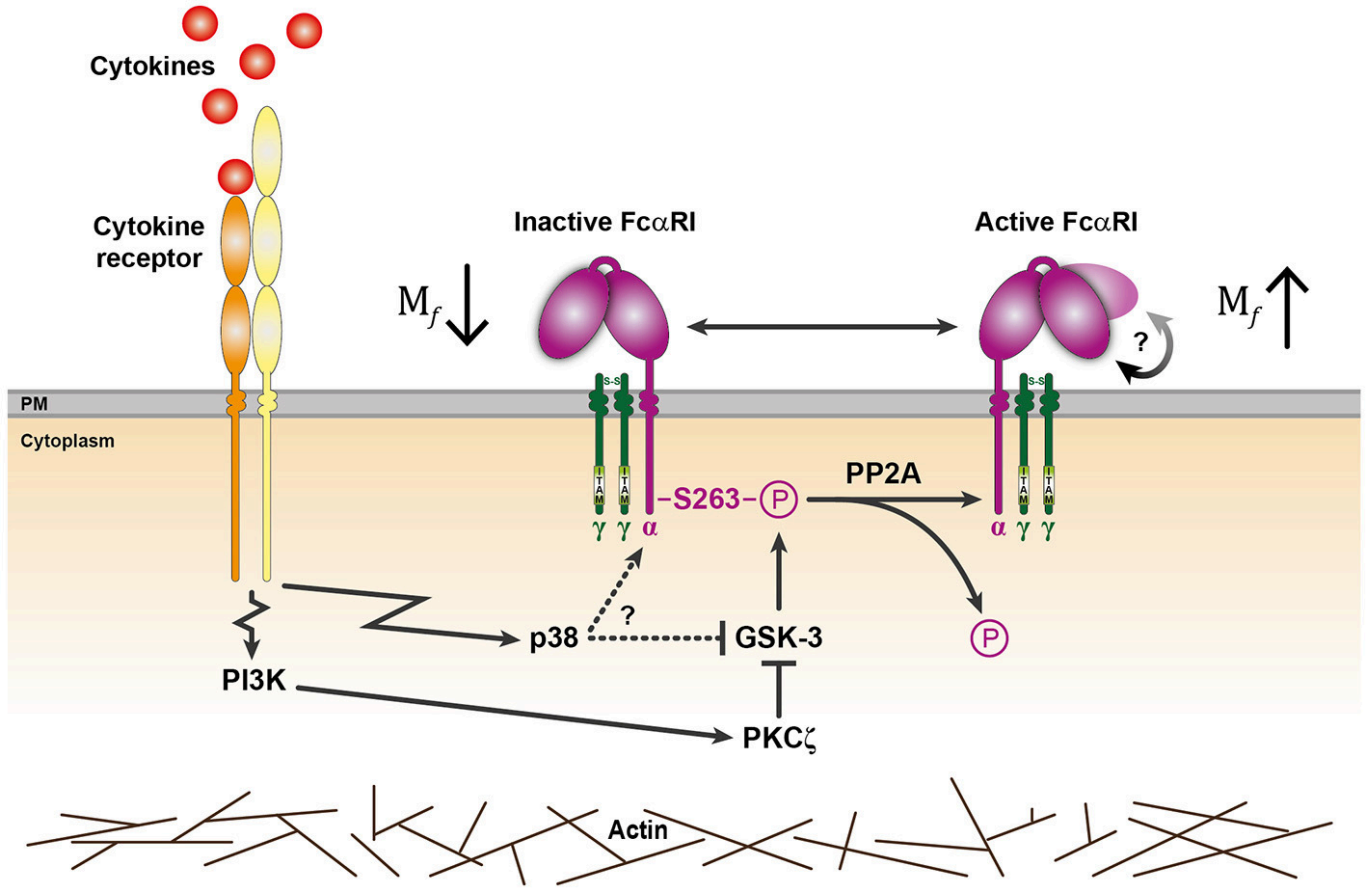
General model for cytokine induced signaling events regulating FcαRI activation. In the absence of stimulation, GSK-3 maintains the inactive state of FcαRI by phosphorylating S263 located in the cytoplasmic tail of the FcαRI α-chain. Binding of cytokines to their receptor can trigger p38 mitogen-activated protein kinase (MAPK) and/or PI3K activation (17, 23). PI3K can activate FcαRI via PKCζ that can phosphorylate and thereby inhibit GSK-3. Possibly, GSK-3 could also be directly inhibited by p38 when activated, but this remains to be established. Upregulated PP2A activity and impairment of GSK-3 leads to FcαRI dephosphorylation and activation. Besides an increase in mobile receptors, a conformational change is possibly involved (indicated by the rounded arrow) that enhances FcαRI affinity. Actin cytoskeleton rearrangements can facilitate the inside-out signaling of these FcαRI.

The mechanism by which cytokine induced inside-out signaling modulates FcαRI function might be at the level of affinity for IgA as well as nano-scale organization of the receptor, which could influence the amount of receptors (valency) available for effective interaction with IgA immune complexes. Therefore, we studied the lateral mobility of FcαRI in the plasma membrane using fluorescence recovery after photobleaching (FRAP) (33). Ba/F3 cells were generated that ectopically express FcαRI fused to yellow fluorescent protein (YFP) to its intracellular tail (Figure 3A). Since it is essential that any cellular movement during FRAP is prevented, we developed a protocol to fix the cells in space using a fibrin matrix. Ba/F3 cells expressing FcαRI-EYFP wt were cytokine starved and subsequently stimulated with or without IL-3. For FRAP, a small square (~1 μm^2^) spanning the plasma membrane was bleached and the recovery of the fluorescence in this region was measured (Supplemental Figure 2, and Figures 3A,B). The recovery of fluorescence intensity was determined at each timepoint after photobleaching relative to the fluorescent intensity before photobleaching. All fluorescent intensities where then averaged, plotted and curve fitting was performed for each condition. FRAP measurements of cytokine starved (*n* = 223) and IL-3 stimulated cells (*n* = 226) revealed that the mobile fraction (*M*_*f*_) of total FcαRI increased from 65.9 ± 0.3% to 74.4 ± 0.2% after stimulation (Figures 3B,C). We continued exploiting FRAP to demonstrate and confirm the involvement of GSK-3 and PP2A in cytokine mediated inside-out signaling. The specific GSK-3 inhibitor CHIR-99021 was anticipated to increase the FcαRI *M*_*f*_ in the absence of cytokine stimulation. Indeed, GSK-3 inhibition results in an increase of FcαRI *M*_*f*_, possibly caused by basal PP2A activity. If our current model presented in Figure 4 is valid, PP2A or PKCζ inhibition should prevent the cytokine induced increase in FcαRI *M*_*f*_. As demonstrated in Figure 3C, both the PP2A inhibitor okadaic acid (OA) and the PKCζ pseudo-substrate prevented the cytokine induced increase of the FcαRI *M*_*f*_. The use of pseudo substrate even resulted in a lower *M*_*f*_ than in unstimulated conditions indicating again the crucial role of this kinase for efficient engagement of FcαRI with IgA opsonized surfaces. We also determined the *M*_*f*_ of the constitutively active FcαRI S263A and inactive S263D mutants in the presence of cytokine stimulation. In Figure 3C, the FcαRI S263D mutant displays an even smaller *M*_*f*_ (60.3 ± 0.4%) compared to the unstimulated FcαRI wt cells. In contrast to the FcαRI S263D, the S263A mutant displays a *M*_*f*_ similar to IL-3 stimulated cells (Figure 3C).

For all conditions tested, the averaged data points were best fitted using a two-phase association curve (Supplemental Figure 3 and according to *F*-test in GraphPad Prism). This implies that FcαRI at the cell surface can be divided into at least two types of mobile fractions that differ in mobility (a fast and a slower moving fraction). Changes in receptor velocity can be revealed by calculating the half-time (t½) of recovery from the acquired fluorescent intensity recovery curves. However, significant changes in t½ of both mobile phases between conditions were never seen in our experiments. Thus, alterations in FcαRI lateral velocities are not observed and seem not involved in the increase of IgA binding after cytokine mediated inside-out signaling.

To confirm the results from our FRAP measurements, we also used fluorescence loss in photobleaching (FLIP). Here, fluorescence intensity at the plasma membrane is monitored on one side of the cell, while the opposite side is bleached with regular intervals (Supplemental Figure 4). The fluorescence of FcαRI-YFP in the non-bleached area is decreasing faster in the presence of IL-3 or when the active mutant (S263A) is used (Figure 3D). This suggest again that the FcαRI mobility is increased upon activation while this can be prevented by an inhibitor of the inside-out signaling pathway like the PKCζ pseudo-substrate (Supplemental Figure 5).

Together, these findings indicate that cytokine induced inside-out activation of FcαRI is associated with an increased amount of mobile receptors that are available for dynamic interactions with IgA immune complexes. This could increase valency and contribute significantly to FcαRI avidity for IgA immune complexes, culminating in efficient signaling and the initiation of cytotoxic cellular effector functions.

## Discussion

Cytokines, such as interleukins, are important regulators of cellular activation of (innate) immune cells. Part of this activation involves the modulation of adhesion receptors, complement receptors and Fc-receptors (FcR). Binding of immunoglobulin-coated targets to FcαRI, FcγRI, and FcγRII is dependent on cytokine stimulation of the cells (3, 11, 12, 14, 23, 34). The cytokine-mediated activation of FcαR relies on the PI3K signaling pathway (17). This concept of inside-out signaling is comparable to the activation mechanisms previously described for integrins, where the cytoplasmic tails of the integrin subunits are targets for the modulation of its affinity/avidity (24, 35).

Upon cytokine induced activation of PI3K and production of PI(3,4,5)P_3_, PDKs are recruited to the membrane. Downstream targets of PDKs include Akt/PKB (18), p70 S6 Kinase (p70S6K) (19), and PKC isoforms, including Ca^2+^ independent PKCs (PKC δ/ε/η), and the atypical PKCζ (20–22). A screen with inhibitors of these kinases (Figure 1) revealed that activation of PKCζ is required for FcαRI activation. This was confirmed in primary cells (Supplemental Figure 1) and by overexpression of a kinase dead PKCζ, which inhibited IgA binding up to 45%, despite the presence of endogenous PKCζ. Identifying PKCζ in the FcαRI activation mechanism also fits with the knowledge that both are Ca^2+^ and DAG independent (22). In addition, the PKCζ pseudo-substrate inhibited the IgA binding to constitutively active PI3K expressing cells, firmly establishing that activation of PKCζ downstream of PI3K is required for IL-3 induced FcαRI activation (Figure 1). Interestingly, it has been described that activation of LFA-1 by chemokines is dependent on PKCζ, suggesting similar activation mechanisms for FcαRI and integrins (36). The p38 MAPK has been implicated in the IL-4 and IL-5 mediated inside-out signaling of FcαRI (23). Furthermore, p38 is reported to directly inhibit GSK-3 activity (37), but whether this event is (partly) regulating FcαRI activation remains to be investigated. It was, however, established that the IL-3 mediated increase of IgA binding by FcαRI transduced Ba/F3 cells does not require p38 activity (17).

FcαRI is not activated in Ba/F3 cells or primary human eosinophils (12) deprived of cytokine stimulation. This suggests a mechanism that constitutively suppresses FcαRI activation in resting cells. Therefore, cytokine induced activation of FcαRI is probably caused by inhibition of this mechanism [see also publications by Bracke et al. (16) and Bakema et al. (15)], allowing them to respond rapidly to a change in their environment. It is tempting to speculate that FcαRI suppression, by phosphorylation on S263, is maintained by a serine/threonine kinase in the absence of cytokine stimulation. The serine/threonine kinase glycogen synthase kinase 3 (GSK-3) is one of the few kinases described to be constitutively active in unstimulated cells, and a reported downstream target of PKCζ (31, 32). Here, we demonstrate that GSK-3 is able to phosphorylate the intracellular tail of FcαRI (Figure 3C) and that its functional disruption results in an increased IgA binding in the absence of IL-3 stimulation (Figure 2A). We also show that GSK-3 itself is phosphorylated in Ba/F3 cells after cytokine stimulation, probably causing its inhibition. As expected, this phosphorylation can be inhibited by the PKCζ pseudo-substrate and by the PI3K inhibitor LY294002 (Figure 3B). GSK-3 is a crucial kinase regulating many cellular processes and is reported to have about 100 substrates (30, 38). To our knowledge this is the first study that identifies FcαRI as a new target of GSK-3. Consensus motifs are identified for many, but not all GSK-3 substrates. The intracellular tail of FcαRI does not contain any of the known motifs for GSK-3 recognition and will require further research.

Interaction of FcαRI with the cytoskeleton might be important in maintaining FcαRI activation. Disruption of the cytoskeleton with cytochalasin D treatment inhibits IgA binding, suggesting that the cytoskeleton contributes to FcαRI activation by stabilizing an active organization (16). It might be possible that the cytokine induced PI3K-PKCζ-GSK-3 pathway affects the cytoskeleton, in addition to (de)phosphorylation of the receptor.

Dynamics in FcαRI behavior at the plasma membrane could determine, at least partly, the avidity switch for IgA. This includes alteration in receptor mobility, velocity, and/or cluster organization within lipid domains. Therefore, the FcαRI lateral mobility was investigated in spatially fixed Ba/F3 cells using FRAP. In Figure 3 we provide evidence that the amount of mobile FcαRI increases when it is (constitutively) activated. In contrast, omitting cytokine stimulation or mimicking the inactive FcαRI by the S263D mutation results in a lower *M*_*f*_. It can be argued that cytokine stimulation of a cell will affect many processes, including membrane dynamics, which would also affect FcαRI mobility. The *M*_*f*_ of the FcαRI S263D in the presence of IL-3 is, however, even lower than observed for the cytokine starved FcαRI wt expressing cells. It is therefore unlikely that an overall activation of the cell is responsible for the increase in FcαRI *M*_*f*_ after cytokine stimulation. The regulatory mechanisms of FcαRI function are believed to be distinct from other FcRs. Therefore, equivalent FRAP experiments were performed using FcγRI-EYFP expressing Ba/F3 cells. Although efficient immune complex binding to FcγRI is also reported to be under inside-out signaling control (14), cytokine stimulation did not affect the *M*_*f*_ of FcγRI (39). This indicates that there is no general inside out signaling pathway dictating ligand binding for all FcR.

The FcαRI *M*_*f*_ was also affected when GSK-3 was modulated in our FRAP experiments, since the use of GSK-3 inhibitor CHIR-99021 resulted in an increase in FcαRI *M*_*f*_ in the absence of cytokine stimulation. Possibly, basal PP2A activity is sufficient to induce this increase. In the presence of the GSK-3 inhibitor CHIR-99021, it might be expected that the *M*_*f*_ would reach a similar level as in the cytokine stimulated cells. The observation that the *M*_*f*_ is smaller than in stimulated cells indicates and confirms that cytokine mediated activation of PP2A is necessary to obtain the maximum amount of mobile receptors (15, 40). In addition, the *M*_*f*_ observed in the presence of OA fits with the notion that PP2A activity is required for achieving an optimal FcαRI organization at the plasma membrane. Results from FRAP measurements in the presence of PKCζ pseudo-substrate are also in line with our earlier observations and model that a decrease in *M*_*f*_ confers poor binding of IgA opsonized surfaces. Next to FRAP, FLIP was used to observe changes in FcαRI behavior at the plasma membrane. This technique visualizes and confirms altered FcαRI mobility, as both cytokine stimulation and the active mutant display a faster decrease of fluorescence during repetitive bleach cycles. In line with our expectations, the PKCζ pseudo-substrate was able to prevent this cytokine induced decrease of fluorescence and displayed similar dynamics as cytokine starved cells (Supplemental Figure 5). Taken together, our findings suggest that activation of FcαRI function is, at least in part, mediated by an increased avidity due to an increased number of mobile receptors.

The differences detected by our FRAP experiments indicate that optimal engagement of FcαRI with IgA involves alterations in receptor dynamics at the cell surface. FcαRI is able to interact with IgA in a 1:1 or a 2:1 (FcαRI:IgA) stoichiometry (41). A bivalent binding of FcαRI to IgA would result in a stronger association. Possibly, cytokine induced inside-out signaling might adjust receptor organization in such a way that bivalent binding is promoted. Next to FcαRI membrane organization, its conformation could be altered upon inside-out signaling. Studies by Herr et al. suggest that FcαRI can acquire different conformations (42). In the case of integrins, conformation specific antibodies were used to help establish low affinity and high affinity conformations (10). Similarly, we generated antibodies against cell surface FcαRI and screened them for specific binding to either FcαRI S263A or FcαRI S263D. Antibodies that were highly specific for either FcαRI mutant were identified, but establishing stable hybridoma clones for further characterization was, unfortunately, unsuccessful (3). This does imply that the extracellular domains of these FcαRI mutants can adopt different conformations.

In summary, we propose a model in which GSK-3 keeps FcαRI in an inactive state by continuously phosphorylating the cytosolic serine residue at position 263. Upon activation with cytokines, a PI3K-PKCζ dependent pathway inhibits GSK-3 and the upregulated serine/threonine phosphatase PP2A dephosphorylates FcαRI, which results in receptor activation (15) (Figure 4). This process exemplifies that leukocytes are tightly regulated and can respond very rapidly and efficiently to their environment. Furthermore, understanding the molecular mechanism behind cytokine induced inside-out signaling of FcαRI will support the development of IgA therapeutics.

## Author Contributions

TtB, HH, MB, LK, and JL: conceptualization. TtB, HH, MB, SJ, JvdL, LK, and JL: methodology. TtB, HH, AB, JB, and DK: formal analysis. TtB, HH, JB, and DK: investigation. TtB and HH: writing—original draft. TtB, JB, AB, LK, and JL: writing—review and editing. TtB, HH, and JB: visualization. LK and JL: supervision. TtB, LK, and JL: funding acquisition.

### Conflict of Interest Statement

The authors declare that the research was conducted in the absence of any commercial or financial relationships that could be construed as a potential conflict of interest.

## Supplementary Material

Supplemental Figure 1 PKCζ pseudo substrate indicates that PKCζ is also involved in cytokine induced FcαRI activation on monocytes. Freshly isolated blood monocytes were first treated for 30 min with 1 μM OA in order to dampen the isolation induced activation of the monocytes (15). Monocytes were then incubated for 15 min in the presence or absence of OA, GM-CSF and/or PKCζ ps as indicated. IgA-coated Dynabeads were then allowed to bind to the adhered monocytes for 10 min, washed, fixed, and quantified for the percentage of rosettes (black bars). A rosette is defined as a cell that has two or more beads bound to it. Like with the Ba/F3 cells, the pseudo substrate is able to inhibit IgA binding despite the presence of cytokine (GM-CSF) stimulation. A minimum of 1,131 total cells or more were counted per condition. For control of background binding, Dynabeads coated with human serum albumin were used (gray bars, NR, no rosettes). A minimum of 700 total cells or more were counted per condition. Overall, very little background binding is observed. Experiment was performed twice and a representative example is shown.

Supplemental Figure 2 Example of a FRAP measurement. Selection of images from a typical FRAP measurement (comprising of 250 images in total) is displayed. The red box indicates the bleach area of the cell boundary (plasma membrane). Between frame 10 and 11 the bleach with high intensity laser light is executed resulting in loss of fluorescence (frame 11) and recovery of fluorescence (frame 12, 13, 25, 30, 40, 70, and 140). Below, raw data FRAP profile of intensities for each time point (frame) are displayed in red by the ZEISS ZEN software.

Supplemental Figure 3 One phase and two phase association curve fitting of FRAP measurements. FcαRI-EYFP wt and S263 mutant expressing Ba/F3 cells were cytokine starved overnight and then incubated with pharmacological inhibitors (CHIR-99021, 5 μM; okadaic acid, 1 μM; PKCζ ps, 10 μM) as indicated. Cells were then stimulated with or without IL-3 before FRAP measurements. Mean values of cells are plotted and one phase (left) and two phase (right) association curve fitting was performed using Graphpad 7.

Supplemental Figure 4 Example of a FLIP measurement. Selection of images from a typical FLIP measurement (comprising of 35 images in total) is displayed. The red box indicates the bleach area of the cell boundary (plasma membrane). After frame 6 (10 s) the indicated plasma membrane area is repetitively bleached with high intensity laser light and the fluorescence loss is monitored in the yellow and light blue plasma membrane regions. It is apparent that the fluorescence intensity in the plasma regions away from the bleached area is gradually decreasing during the course of the measurement. Fluorescence intensity of a neighboring cell (green region) remains relatively stable and is used for correcting the FLIP measurement in the analysis. Below, raw data of fluorescence intensities per region for each time point (frame) are displayed by the ZEISS ZEN software.

Supplemental Figure 5 FLIP measurements of FcαRI-YFP in the absence or presence of IL-3 and PKCζ ps. FcαRI-EYFP wt expressing Ba/F3 cells were cytokine starved overnight and then pre-incubated with or without the PKCζ ps (10 μM) for 15 min to interfere with PKCζ function. Cells were then stimulated with or without IL-3 before FLIP measurements. Mean of corrected and normalized fluorescence values (±SEM) of cells pooled from three experiments are plotted and one phase association curve fitting was performed using Graphpad 7. Average fluorescence of six images (frame 1 through frame 6) before the start of bleach cycles was set at 100%. For the no IL-3 condition 44 measurements, for the +IL-3 condition 32 measurements and for the +IL-3 +PKCζ ps condition 24 measurements were included.
