# Supplementary material for: FcαRI Dynamics Are Regulated by GSK-3 and PKCζ During Cytokine Mediated Inside-Out Signaling
Source: Front Immunol. 2019 Jan 31;9:3191. doi: 10.3389/fimmu.2018.03191 (PMC6365424; doi:10.3389/fimmu.2018.03191)

Supplemental figure 1

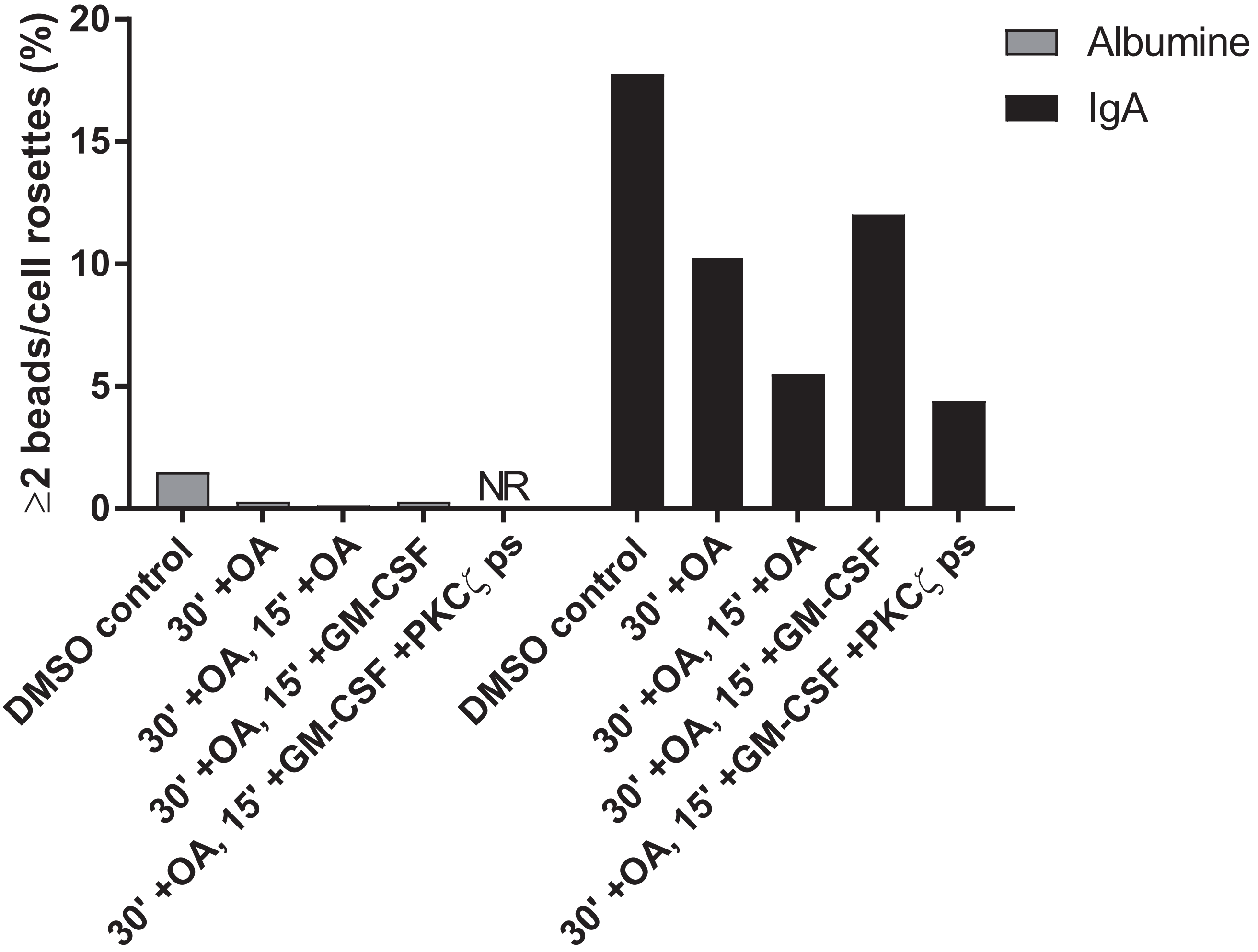

Supplemental figure 2

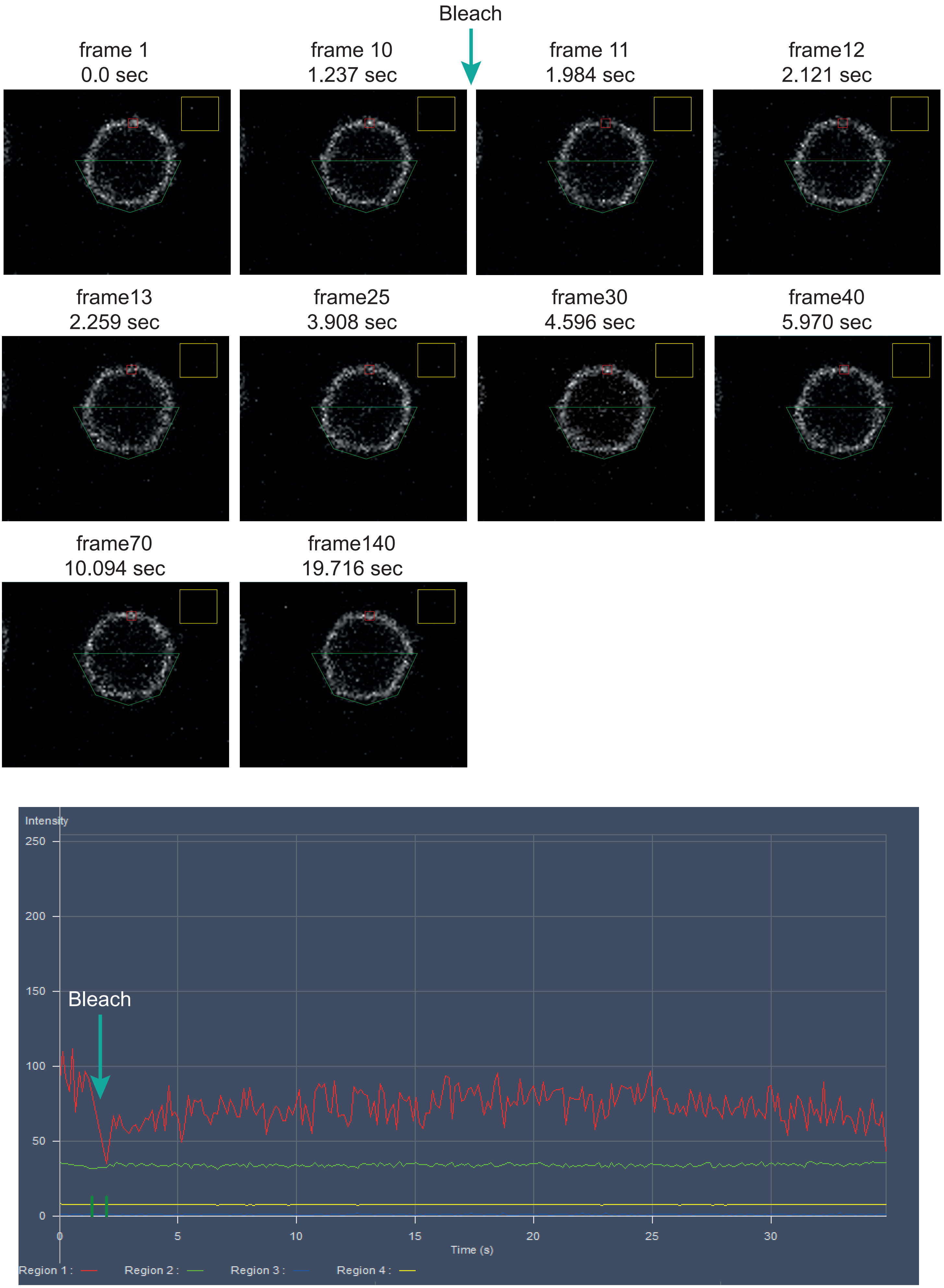

Supplemental figure 3

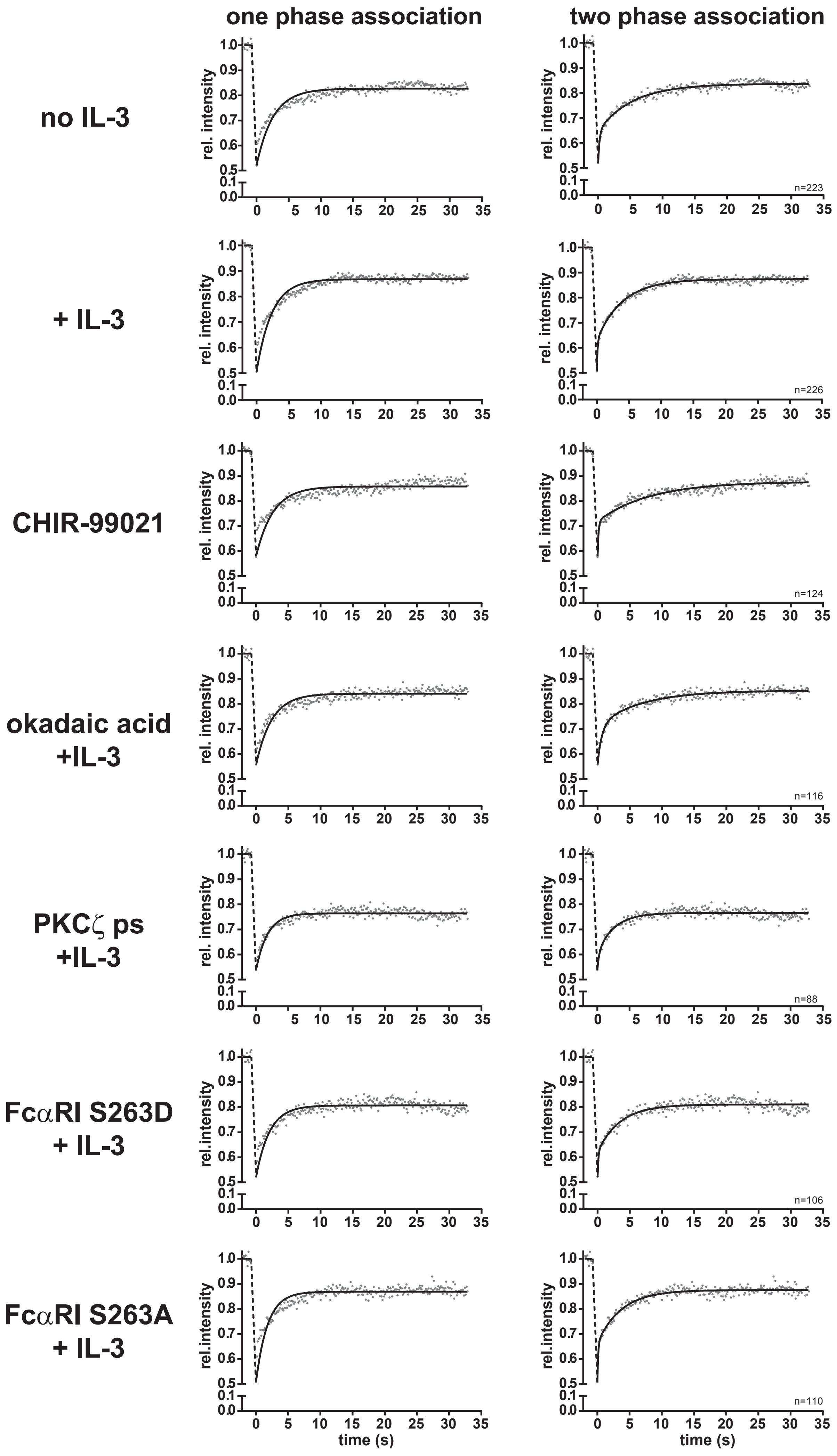

Supplemental figure 4

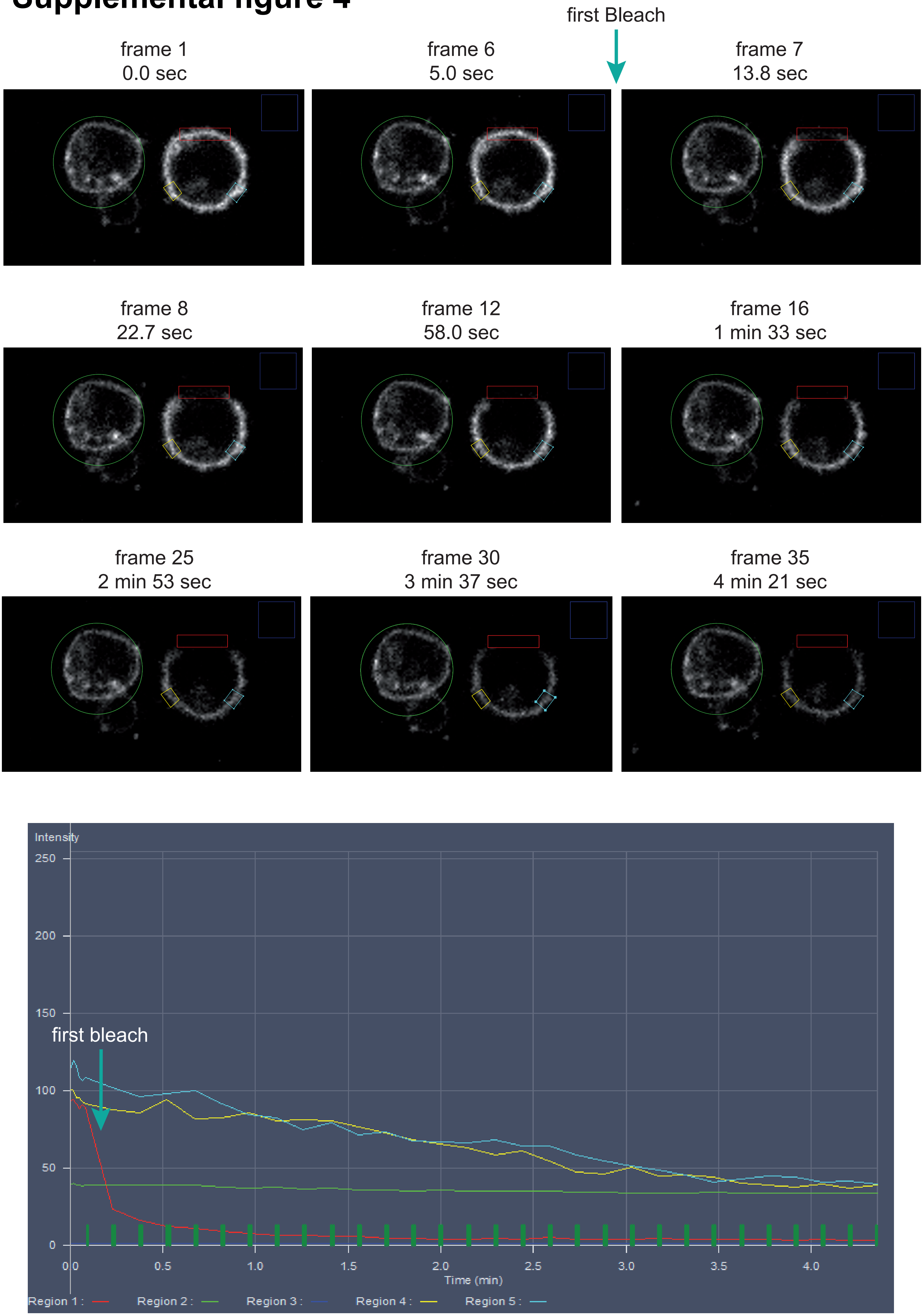

Supplemental figure 5

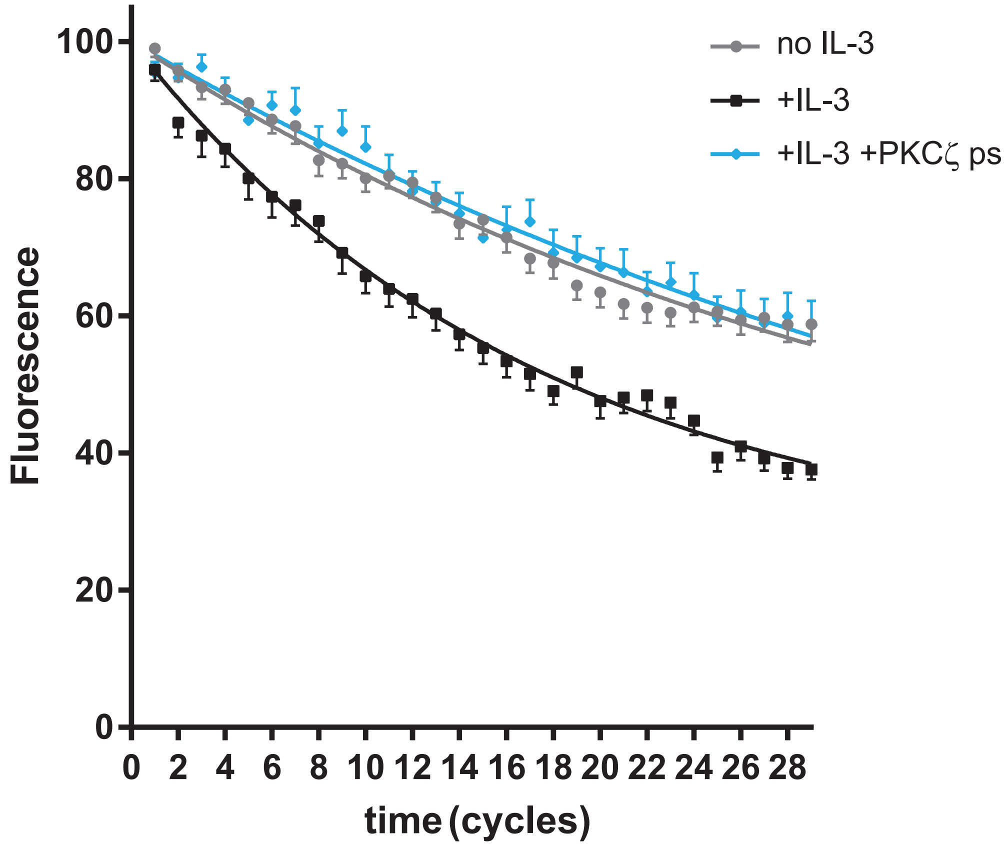

Supplement: Supplemental Figure 1 — PKCζ pseudo substrate indicates that PKCζ is also involved in cytokine induced FcαRI activation on monocytes. Freshly isolated blood monocytes were first treated for 30 min with 1 μM OA in order to dampen the isolation induced activation of the monocytes (15). Monocytes were then incubated for 15 min in the presence or absence of OA, GM-CSF and/or PKCζ ps as indicated. IgA-coated Dynabeads were then allowed to bind to the adhered monocytes for 10 min, washed, fixed, and quantified for the percentage of rosettes (black bars). A rosette is defined as a cell that has two or more beads bound to it. Like with the Ba/F3 cells, the pseudo substrate is able to inhibit IgA binding despite the presence of cytokine (GM-CSF) stimulation. A minimum of 1,131 total cells or more were counted per condition. For control of background binding, Dynabeads coated with human serum albumin were used (gray bars, NR, no rosettes). A minimum of 700 total cells or more were counted per condition. Overall, very little background binding is observed. Experiment was performed twice and a representative example is shown. [file Data_Sheet_1.PDF]
